# The effect of a prior eccentric lowering phase on concentric neuromechanics during multiple joint resistance exercise in older adults

**DOI:** 10.1111/sms.14435

**Published:** 2023-06-22

**Authors:** Emmet J. Mc Dermott, Thomas G. Balshaw, Katherine Brooke‐Wavell, Thomas M. Maden‐Wilkinson, Jonathan P. Folland

**Affiliations:** ^1^ Versus Arthritis, Centre for Sport, Exercise and Osteoarthritis Research Loughborough University Leicestershire UK; ^2^ School of Sport, Exercise and Health Sciences Loughborough University Leicestershire UK; ^3^ Department of Engineering, School of Science and Technology Nottingham Trent University Nottinghamshire UK; ^4^ Physical Activity, Wellness and Public Health Research Group, Department of Sport and Physical Activity, Faculty of Health and Wellbeing, Collegiate Campus Sheffield Hallam University Sheffield UK

**Keywords:** aging, muscle activation, neuromechanics, resistance training prescription

## Abstract

Aging involves a marked decline in physical function and especially muscle power. Thus, optimal resistance exercise (RE) to improve muscle power is required for exercise prescription. An eccentric lowering phase immediately before a concentric lift (ECC‐CON) may augment concentric power production, due to various proposed mechanisms (e.g., elastic recoil, pre‐activation, stretch reflex, contractile history), when compared with a concentric contraction alone (CON‐Only). This study compared the effect of a prior eccentric lowering phase on older adult concentric power performance (ECC‐CON vs. CON‐Only) during a common multiple joint isoinertial RE (i.e., leg press) with a range of loads. Twelve healthy older adult males completed two measurement sessions, consisting of ECC‐CON and CON‐Only contractions, performed in a counterbalanced order using 20–80% of one repetition maximum [% 1RM] loads on an instrumented isoinertial leg press dynamometer that measured power, force, and velocity. Muscle activation was assessed with surface electromyography (sEMG). For mean power ECC‐CON>CON‐Only, with a pronounced effect of load on the augmentation of power by ECC‐CON (+19 to +55%, 35–80% 1RM, all *p* < 0.032). Similarly, for mean velocity ECC‐CON>CON‐Only, especially as load increased (+15 to 54%, 20–80% 1RM, all *p* < 0.005), but mean force showed more modest benefits of ECC‐CON (+9 to 14%, 50–80% 1RM, all *p* < 0.05). In contrast, peak power and velocity were similar for ECC‐CON and CON‐Only with all loads. Knee and hip extensor sEMG were similar for both types of contractions. In conclusion, ECC‐CON contractions produced greater power, and velocity performance in older adults than CON‐Only and may provide a superior stimulus for chronic power development.

## 1 INTRODUCTION

As people age, muscle power invariably declines^1^ with low leg muscle power associated with increased fall risk,^2^ loss of mobility and impaired functional independence of older adults.^3^ Similarly, in osteoarthritis, one of the most common age‐related musculoskeletal conditions, power has also been found to be a better predictor, than strength, of whole‐body physical function,^4^ self‐reported function,^5^ knee joint mechanics,^6^ pain and quality of life.^7^ To offset this age induced change in muscle power, it is widely recommended that older adults perform regular resistance exercise (RE), which typically involves isoinertial contractions, lifting and lowering a fixed mass.^8^, ^9^ However, our understanding of how to maximize power production during the RE of older adults is incomplete and could limit the stimulus for power development with regular training.

For older adults performing RE, we recently found that fast ballistic contractions (i.e., where the load is projected/thrown) generated superior neuromuscular power compared with both conventional and slow controlled, as well as fast but non‐ballistic (i.e., the load is not projected/thrown), contractions during the concentric phase of a lift.^10^ Given the higher power production of fast ballistic contractions they may provide a more potent stimulus for power development with regular RE.^10^, ^11^ However, in our previous experiment fast, ballistic concentric contractions were performed from rest immediately before the concentric phase of the movement (CON‐Only). RE can be performed with a deliberate rest between repetitions (i.e., unloading that results in CON‐Only lift) or as a continuous movement with a lowering eccentric phase immediately preceding a concentric lifting phase (ECC‐CON, i.e., a stretch shortening cycle).^8^ It is possible that power production during fast ballistic concentric contractions may be further enhanced by performing an eccentric lowering phase immediately prior to the concentric lift^12^ and, thus, further optimize the stimulus for power development. Interestingly, in young adults a prior eccentric phase has generally been shown to produce greater concentric power, force and velocity performance than CON‐Only during upper (i.e., bench press) and lower body (i.e., squat and countermovement jump) RE,^13^, ^14^, ^15^, ^16^, ^17^, ^18^ with these mechanical differences underpinned by greater muscle activation during the concentric phase of ECC‐CON versus CON‐Only contractions.^15^ However, some contradictory findings have also been reported, with no differences in power, force, velocity performance^19^ or muscle activation^20^, ^21^ between ECC‐CON and CON‐Only contractions in young adults. Several mechanisms have been suggested to enhance concentric phase performance (e.g. power, force and velocity) of ECC‐CON versus CON‐Only.^22^ Firstly, energy stored by the parallel and series elastic components during the prior eccentric action and subsequently released during the concentric motion may enhance concentric performance.^23^, ^24^, ^25^ Second, pre‐activation during the eccentric phase that provides a high active force state at the onset of concentric action, may minimize the delay in achieving full activation and the need to remove the slack from the muscle‐tendon‐unit during the concentric phase, thus enhancing concentric function.^26^, ^27^, ^28^ Thirdly, a stretch reflex response to the prior eccentric contraction could enhance concentric phase neural activation and performance.^29^ Finally, the prior eccentric action may provoke a residual force enhancement (RFE) at the sarcomere level that could persist during the subsequent concentric action.^30^

Moreover, in older adults, the potential neuromechanical benefits of a prior eccentric phase immediately prior to concentric contraction could be particularly advantageous for concentric power production, but this possibility has not been investigated. Older adults experience a greater decline in concentric neuromuscular power, than isometric strength,^1^ which may be, in part, due to their greater shortening‐induced force depression.^31^ In contrast, older adults appear to preserve eccentric strength better than concentric or isometric function,^32^ in part because they also experience greater RFE with eccentric contractions.^24^ A stretch‐shortening cycle (ECC‐CON) contraction in older adults may, thus, use their disproportionately high eccentric performance and RFE to facilitate their otherwise relatively poor concentric performance. However, the capacity of a prior eccentric contraction to augment the concentric power, force, and velocity performance of older adults during RE remains unknown.

Furthermore, the greatest advantage of the prior eccentric contraction could occur at the start of the concentric phase due to prior activation and force compared with CON‐Only starting from rest (i.e., zero activation and force^12^, ^17^). Whether this advantage of ECC‐CON is short lived or persists throughout the concentric phase of contraction is currently unclear (see earlier discussion of the somewhat contrary findings in the literature). The extent of the advantage provided by ECC‐CON may also be dependent on the load lifted as higher RT loads likely involve greater force and activation during the eccentric phase and, thus, provide a greater advantage at the onset of the concentric phase compared with CON‐Only. On the other hand, the more prolonged contraction with a prior eccentric phase could lead to some fatigue perhaps favoring CON‐Only during the later phase of concentric action, and this could have contributed to the contradictory findings in the literature. In any case, potential differences between ECC‐CON and CON‐Only may be informed by measuring activation, force, velocity, and power throughout the entire concentric phase of contraction.

The primary aim of this study was to determine if an eccentric lowering phase immediately prior to the concentric phase (ECC‐CON) accentuates concentric power production compared with starting the concentric phase from rest (CON‐Only) during fast, ballistic RE in healthy older men with the wide range of loads typically used for RE (20, 35, 50, 65, and 80% of one repetition maximum [% 1RM]). Secondary aims included assessment of the underlying determinants of power: force, velocity, and muscle activation (assessed with surface electromyography [sEMG]). Mechanical variables were expressed as mean, peak, and instantaneous values throughout contraction to comprehensively compare the different types of contractions. It was hypothesized that ECC‐CON contractions would generate greater mean power with all loads than CON‐Only contractions.

## 2 MATERIALS AND METHODS

### 2.1 Participants

Twelve older men (age: 66 ± 5 years; height: 1.81 ± 0.10 m; body mass: 78.5 ± 11.0 kg; BMI: 24.0 ± 2.7 kg·m^−2^) volunteered to participate and provided written informed consent before completing this study that was approved by the Loughborough University Ethics Approval (Human Participants) Sub‐Committee. Participants were recreationally active with a low to moderate level of mainly aerobic physical activity (2175 ± 1450 MET·min·week; e.g., walking, running, and cycling). Inclusion criteria were as follows: no recent (previous 6 months) history of moderate or severe lower extremity musculoskeletal injury; no history of major surgery, musculoskeletal or in the involved leg or neuromuscular disease; no medical conditions warranting exclusion from exercise; and a BMI > 28 kg·m^−2^. Participants were excluded if they: scored <23 on the mini‐mental state exam^33^; had blood pressure of >150/90 mmHg^7^ and took >15 s to complete the sit‐to‐stand test.^34^ Physical activity was assessed using the International Physical Activity Questionnaire (IPAQ, short form).^35^

### 2.2 Experimental design

All participants visited the neuromuscular function laboratory on three separate occasions: one familiarization session and two measurement sessions (5–10 days apart). All measurement sessions were conducted at a consistent time of day, commencing between 12:00 and 18:00, and involved unilateral leg press contractions with an instrumented isoinertial leg press dynamometer for recording of force and displacement, that facilitated the derivation of velocity and power (see below). Each measurement session started with preliminary isometric maximum voluntary contractions (MVCs; for normalization of sEMG) before participants performed contractions with a range of progressively higher loads during both measurement sessions (session one, 20, 35, 50% 1RM; session two, 50, 65 & 80% 1RM) using both types of contractions with each load before moving to the next load. The order of the types of contractions was counterbalanced (i.e., half the participants did CON‐Only then ECC‐CON during session 1 and the reverse during session 2). The dominant leg (n = 10) was assessed unilaterally except (n = 2; non‐dominant leg) when there was a history of dominant leg injury. The familiarization session involved preliminary measurements, that is, one repetition maximum (1RM) for load prescription during subsequent sessions, practice of the MVCs and 4–5 practice efforts with both types of contractions at each of two loads, 20 and 65% 1RM.

### 2.3 Kinetic, kinematic, and surface electromyography recordings

Participants were seated on the leg press dynamometer, with a fixed seat and adjustable (40 mm canvas webbing) straps used to restrain the pelvis and prevent extraneous movement. The dynamometer enabled measurements during a leg press action (simultaneous hip extension, knee extension and plantar flexion), with the participant “pressing” against a plate loaded sled on a linear low friction track (30° inclined, as previously described, see Ref. [11]). The sled was instrumented with a bespoke calibrated force plate (Force Logic, Swallowfield, UK) consisting of four single axis load cells (CDC, model SP 3949; each 2 kN capacity; total capacity = 8 kN) in a parallel rectangular formation (load cell spacing: length [0.25] × width [0.14 m]) secured between two aluminium plates that were attached to the original foot plate of the sled and perpendicular to the sled track. Following extensive pilot work, to reduce ankle dorsiflexion and associated discomfort at the start of the leg press movement (common in older adults) a further modification involved mounting a rigid aluminium wedge on the force plate to provide a surface foot plate at an angle of 21° to the force plate (surface area, 0.36 × 0.23 m). The leg‐press dynamometer was constructed with multiple one‐way adjustable mechanical catches, that effectively “caught” the loaded sled once projected, to facilitate safe projection of the sled during ballistic contractions.

For all contractions, the participants' foot position was standardized/replicated in a central position on the surface foot plate using tape markers. For isometric MVCs and passive limb weight measurements only, the participants foot was secured to the surface foot plate using a bespoke foot brace and adjustable strapping, that ensured no active force application and, therefore, a relaxed rested state. A calibrated draw‐wire transducer (WDS‐2500‐P96‐SR‐U, Micro‐epsilon Ltd, Ortenburg, Germany) was used to assess displacement of the sled, with the spindle housing bolted to the static frame of the dynamometer, and the draw‐wire attached, and parallel to, the movement of the sled. The analogue force and displacement signals were sampled at 2028 Hz using an external analogue to digital (A/D) converter (1401 Power 3, CED Ltd., Cambridge, UK), and recorded using Spike 2 computer software (CED Ltd., Cambridge, UK) on a personal computer.

Following the palpation and marking of the muscle borders and electrode positions, the skin was prepared by shaving, abrading, and cleansing (70% ethanol). sEMG was recorded using a wireless EMG system (Trigno; Delsys Inc, Boston, MA, USA) with single differential Trigno sensors (inter‐electrode distance = 1 cm) attached to the skin using an adhesive interface. sEMG sensors were positioned parallel to the presumed orientation of the muscle fibers. Two sEMG sensors were placed on each of the superficial quadricep muscles (rectus femoris [RF], vastus lateralis [VL], vastus medialis [VM]), and positioned relative (%) to thigh length (greater trochanter to knee‐joint space) measured from the superior aspect of the patella: RF_PROXIMAL_ (65%) and RF_DISTAL_ (55%), VL_PROXIMAL_ (55%) and VL_DISTAL_ (50%), and VM_PROXIMAL_ (35%) and VM_DISTAL_ (30%). Single sEMG sensors were placed on each of the following superficial muscles: hamstrings (bicep femoris [BF], medial hamstring [MH]), gastrocnemius (lateral gastrocnemius [LG] and medial gastrocnemius [MG]), soleus (SO), and the gluteus maximus [GM]. Specific locations were as follows: BF and MH both at 45% of thigh length measured from the popliteal fossa; MG and LG at 75% and 85% of shank length (lateral malleolus to knee‐joint space) measured from the calcaneus, respectively; SO at 66% shank length measured from the medial femoral condyle; and GM at 50% of the distance between the second sacral vertebrae and the greater trochanter. The raw sEMG signals were amplified at source (×300; 20–450 Hz) before further amplification (overall total amplification ×909). The sEMG signal was sampled at 2028 Hz via the same A/D convertor as the force and displacement signals. To account for the inherent 48 ms delay present in the Delsys Trigno system, signals were time aligned during offline analysis.

### 2.4 Familiarization session and preliminary measurements

**FIGURE 1:**
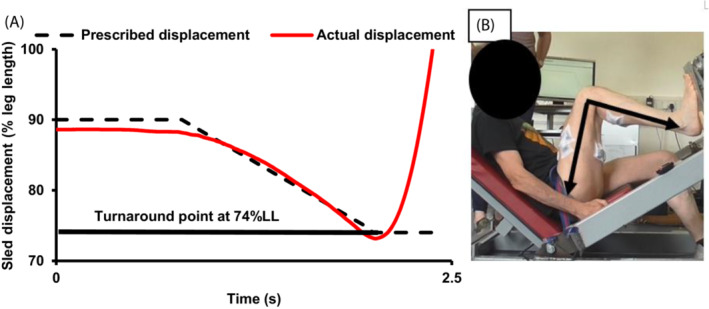
(A) Example of the target lowering displacement–time profile (90–74% of leg length over 1.2 s) presented to participants before each ECC‐CON contraction (dotted line) and an example of the real‐time displacement recording provided for feedback (solid red line) and (B) An example video image of one participant's leg at the ~74% LL position (start of CON‐Only and turnaround point for ECC‐CON), which was equated to a knee joint angle of ~90°.

Familiarization sessions first involved preliminary measurements of leg length, followed by participant's practicing the isometric MVCs according to an identical protocol as for the measurement sessions (see below), that was followed by measurements of passive limb weight, and then 1RM lifting strength, in this order. Finally, participants performed 4–5 practice efforts with both types of ballistic contractions (CON‐Only and ECC‐CON) with each of two loads, 20 and 65% 1RM. Prior to the loaded practice ECC‐CON contractions, participants used a light load (the unloaded sled) to become familiar first with the prescribed lowering duration/rate (matching a target displacement‐time line) and then also the “turnaround point” (indicated by a horizontal line at 74% of leg length [% LL]; see Figure 1A). Once the participants were proficient at matching both the target displacement‐time line and the prescribed turnaround point, they progressed to performing the loaded practice ECC‐CON contractions.

### 2.5 Leg‐length and passive limb weight

During familiarization an individual reference position of full leg length (100% of leg length [% LL]) was determined on the leg press dynamometer. The draw‐wire displacement transducer measurement was noted with the knee fully extended, the leg relaxed and parallel to the sled track, and the plantar surface of the foot flat and central on the surface foot plate. This reference position was used to prescribe individualized isometric measurement positions and for normalization of measurements throughout the range of motion during the isoinertial contractions, that is, to % LL. Finally, with the sled fixed (i.e., stationary) in four different positions (95, 88, 81, and 74% LL) the passive limb weight exerted by the leg on the force plate (i.e., when relaxed and not contracting) was recorded and plotted against displacement to generate a quadratic function. This facilitated the interpolation of passive force at all positions throughout the range of motion for gravity correction during the isoinertial contractions.

### 2.6 One repetition maximum

During familiarization, each participant's concentric leg press 1RM, was determined and used for load prescription during the main measurement sessions. Participants performed preliminary lifts: two at light loads (~ 40–50 kg), and a single lift with a moderate load (1.3 × body mass; ~ 80% 1RM), with ~30 s rest between contractions. Thereafter, a series of near maximum lifts was undertaken to establish 1RM, with the load increased by ~2.5–5 kg after each successful lift. Each lift began stationary at a position of 74% LL and a successful lift was defined by the participant's ability to move the load through the specified displacement (74–95% LL). After each concentric lift, the load was lowered to the start position (74% LL) by the researchers. 1RM was defined as the highest load that could be lifted through the specified displacement range, usually determined within 4–6 attempts, with each maximal attempt interspersed with ≥2 min of recovery.

### 2.7 Measurement Session

All measurement sessions were performed in the following order.

#### 2.7.1 Isometric maximum voluntary contractions

Isometric MVCs were performed to generate reference sEMG values for normalization of sEMG during the isoinertial contractions. Participants performed a standardized isometric warm‐up at one position (95% LL: 3 × 50%, 3 × 75% and 1 × 90% of perceived maximum exertion), with each contraction lasting ~3–5 s. Participants then performed 3–4 MVCs at each of two positions (95 then 74% LL). During each MVC participants were instructed to push as hard as possible for ~3–5 s, with 30 s rest between contractions and 2 min rest between positions. Biofeedback was provided with the force‐time recording displayed prominently in front of the participant and a cursor used to indicate the highest force achieved during that series of MVCs.

During off‐line analysis, sEMG amplitude was assessed as root mean square (RMS) amplitude during a 500 ms epoch (250 ms either side) at isometric maximal voluntary force (iMVF, the highest single instantaneous force at each position). The RMS amplitude of each signal (recording site) was then baseline corrected (i.e., resting RMS amplitude was subtracted). The isometric position that produced the highest RMS amplitude for each functional muscle group (knee extensor [KE_EMG_] 74% LL; hip extensor [HE_EMG_] and plantar flexor [PF_EMG_], 95% LL) was used for normalization of that muscle group during the isoinertial contractions.

#### 2.7.2 Isoinertial contraction protocol and analysis

Participants performed 4–5 maximum effort contractions of each type (CON‐Only and ECC‐CON) with each load before moving to the next load (ascending order: session one = 20, 35, 50% 1RM; session two = 50, 65 and 80% 1RM), with 30 s rest between contractions and ≥2 min between loads. During CON‐Only contractions the participants were instructed to perform the concentric action from rest “as fast as possible” throughout the entire concentric lifting phase, with the load thrown/projected as far as possible. Following each CON‐Only contraction, the investigators lowered the load to the start position at 74% LL. Whereas, for the ECC‐CON contractions participants started the contraction at 90% LL and were instructed to perform an eccentric lowering phase aiming to match a prescribed lowering velocity (90–74% LL in 1.2 s) displayed on a monitor 1 m from the participants for 5 s in advance of the contraction. Then the real‐time displacement was overlaid on the monitor to provide feedback on the correct lowering velocity with the lowest displacement of the ECC‐CON contraction (i.e., the “turnaround point” at 74% LL) also shown on‐screen with a horizontal line (Figure 1A). After the controlled lowering phase, the participant was instructed to rapidly change direction at the “turnaround point” and then perform the concentric phase of the movement “as fast as possible,” with the load thrown/projected as far as possible. Analysis of sagittal plane video collected during pilot work (n = 11) showed the 74% LL turnaround position (start of CON‐Only and turnaround point of ECC‐CON) was equivalent to a knee joint angle of 90.5 ± 3.1° (see Figure 1B).

During offline analysis, force and displacement signals were filtered using a low‐pass fourth‐order zero‐lag Butterworth filter with a cut‐off frequency of 30 Hz. The filtered displacement signal was used to derive velocity (time constant = 15 ms). Force data were gravity corrected by subtracting passive force due to the passive weight of the limb measured statically (see above), to derive active force as the criterion measure of force. Instantaneous external power was calculated as the product of active force and velocity measurements (*P* = F × V). ECC‐CON contractions where the turnaround point (lowest displacement) was >3% LL from the target “turnaround point” of 74% LL (i.e., outside the range 71–77% LL) were discarded from the analysis. The two contractions with highest instantaneous peak power at each load and type of contraction were selected for further analysis with all measurements averaged across these two contractions. For the 50% 1RM load, the two best contractions in each measurement session were also averaged across both measurement sessions.

Peak power, peak force, and peak velocity were determined as the highest instantaneous values measured during the concentric phase of contraction. Mean power, mean force, and mean velocity were averaged throughout the concentric phase of contraction. During CON‐Only the start of the concentric phase of contraction was defined as the point the displacement signal increased above the baseline noise envelope (assessed over 300 ms prior to displacement onset) and did not return, and the end of the concentric lifting phase was defined as follows: force offset (active force <0) when the load was projected/thrown; or at higher loads peak displacement (the highest instantaneous displacement) when the load was not thrown. During the ECC‐CON contractions, the onset of the concentric phase was defined as the minimum displacement. To ensure both ECC‐CON and CON‐Only were compared over a similar range of motion (see Table 1), the lifting range during CON‐Only was added to the ECC‐CON concentric displacement onset. Work done during each contraction was calculated by multiplying mean power by movement duration (see above). Finally, power, force, and velocity were measured at specific percentages of time (10% increments, 0–100%) throughout each analyzed contraction. During pilot work, we considered using either displacement‐ or time‐based measurements throughout contractions. However, displacement‐based measurements were skewed to the later phase of each contraction/lift due to the time taken to increase force and overcome the inertia at the start of the lift, with the first 10% of displacement taking 30–40% of contraction duration. Time‐based increments are also more consistent with the measurement of mean values averaged over time.

**TABLE 1:** Concentric phase range of motion (m), duration (s), work (J) during concentric‐only (CON‐Only) and eccentric‐concentric (ECC‐CON) contractions, as well as the velocity during the eccentric lowering phase of ECC‐CON, with a range of five loads (20–80% 1RM).

	LOAD (% 1RM)				
	20	35	50	65	80
Range of motion (m)					
CON‐Only	0.33 ± 0.03	0.34 ± 0.02	0.35 ± 0.02	0.36 ± 0.03	0.37 ± 0.05
ECC‐CON	0.33 ± 0.03	0.34 ± 0.02	0.35 ± 0.02	0.36 ± 0.03	0.36 ± 0.05
Duration (s)					
CON‐Only	0.33 ± 0.04	0.45 ± 0.04	0.60 ± 0.05	0.81 ± 0.05	1.26 ± 0.32
ECC‐CON	0.29 ± 0.03	0.38 ± 0.03^a^	0.48 ± 0.03^a^	0.59 ± 0.05^a^	0.80 ± 0.11^a^
Work (J)					
CON‐Only	126 ± 29	170 ± 45	215 ± 37	249 ± 45	272 ± 56
ECC‐CON	120 ± 30	177 ± 41	213 ± 41	252 ± 51	269 ± 53
ECC‐CON lowering					
Velocity (m·s^−1^)	−0.21 ± 0.03	−0.23 ± 0.03	−0.21 ± 0.04	−0.20 ± 0.03	−0.19 ± 0.03

*Note*: Data are presented as the mean ± SD.

^a^ Significantly (*p* < 0.05) different to CON‐Only.

During isoinertial contractions, the RMS amplitude of each sEMG signal was measured throughout the concentric phase of contraction as described above. The RMS amplitude of each signal (recording site) was baseline corrected (i.e., resting RMS amplitude was subtracted) and normalized (%) to RMS amplitude at iMVF (knee extensors sites 74% LL; hip extensors and plantar flexors sites 95% LL). The normalized values at each sEMG electrode site were averaged across the two selected contractions at each load (see above), before averaging across sites to produce functional muscle group values (KE_EMG_ = [RF_PROXIMAL_ + RF_DISTAL_ + VL_PROXIMAL_ + VL_DISTAL_ + VM_PROXIMAL_ + VM_DISTAL_]/6; HE_EMG_ = [GM + BF + MH]/3; PF_EMG_ = [SO + MG + LG]/3). Similar to the mechanical variables above, KE_EMG,_ HE_EMG_ and PF_EMG_ were also assessed using a 100 ms epoch (50 ms each side) and normalized to (%) to RMS amplitude at iMVF as previously described and presented at specific percentages of time (10% increments, 0–100%) throughout each analyzed contraction (see supplementary material, Figures S1–S3).

Based on the repeated measurement sessions with 50% 1RM load between‐session measurement reliability (i.e., coefficient of variation [CV]) for each type of contraction (CON‐Only and ECC‐CON) was calculated as peak: power 3.5, 4.9%, force 1.7, 3.2%, velocity 2.3, 2.8%; and mean: power 5.4, 9.7%, force 3.1, 6.5%, and velocity 4.8, 3.9%, respectively. CV values for the functional muscle group activation variables were KE_EMG_ 7.7, 11.6%, HE_EMG_ 17.2, 17.7%, and PF_EMG_ 28.1, 27.2%, respectively.

### 2.8 Statistical analysis

Group data are presented as mean ± SD. Statistical analysis was conducted using SPSS Version 23 (IBM Corp., Armonk, NY) software and the statistical significance was defined as *p* < 0.05. Two factor repeated measures ANOVA was used to assess the effect of contraction type ([CON‐Only and ECC‐CON] and load [20, 35, 50, 65 and 80% 1RM]) on peak and mean measures of force, velocity, power, sEMG amplitude of the three functional muscle groups, and also concentric phase range of motion, duration and work. A further two‐factor repeated‐measures ANOVA was used to assess the effect of contraction type [CON‐Only and ECC‐CON] and percentage contraction duration [0, 10, 20, 30, 40, 50, 60, 70, 80, 90, and 100% of contraction duration] on force, velocity, and power, at each load. Where a significant (*p* < 0.05) main effect of contraction type was found a paired *t*‐test (CON‐Only and ECC‐CON) was performed at each load or percentage of contraction duration, with a stepwise correction for multiple comparisons performed on the paired *t*‐test values to avoid a type 1 error. A one‐way ANOVA was performed to compare the mean eccentric lowering velocity during ECC‐CON contractions across loads, that is, 20, 35, 50, 65, and 80% 1RM. Percentage difference (∆) was calculated ([mean^1^ – mean^2^]/mean^1^*100). Effect sizes (ES; Cohen's d) were calculated for peak and mean kinetic, kinematic and sEMG values using a pooled standard deviation with ES of <0.2 “trivial,” ≥0.2 to ≤0.49 “small,” ≥0.5 to ≤0.79 “moderate,” and ≥0.8 “large.”

## 3 RESULTS

### 3.1 Contraction descriptors

There was an overall main effect of a prior eccentric lowering phase on concentric contraction duration (two‐way ANOVA; all *p* < 0.01, Table 1), specifically with ECC‐CON found to have a shorter contraction duration than CON‐Only with all but the lightest load (16 to 37% shorter with 35–80% 1RM). There was no difference in the work done between ECC‐CON and CON‐Only at any load (two‐way ANOVA, *p* > 0.05). There was no difference between the range of movement for CON‐Only and ECC‐CON (two‐way ANOVA, *p* = 0.201). During ECC‐CON contractions, the mean eccentric lowering velocity appeared consistent and, thus, well controlled across all five loads (one‐way ANOVA; *p* = 0.216).

### 3.2 Concentric phase peak and mean power, force and velocity values

**FIGURE 2:**
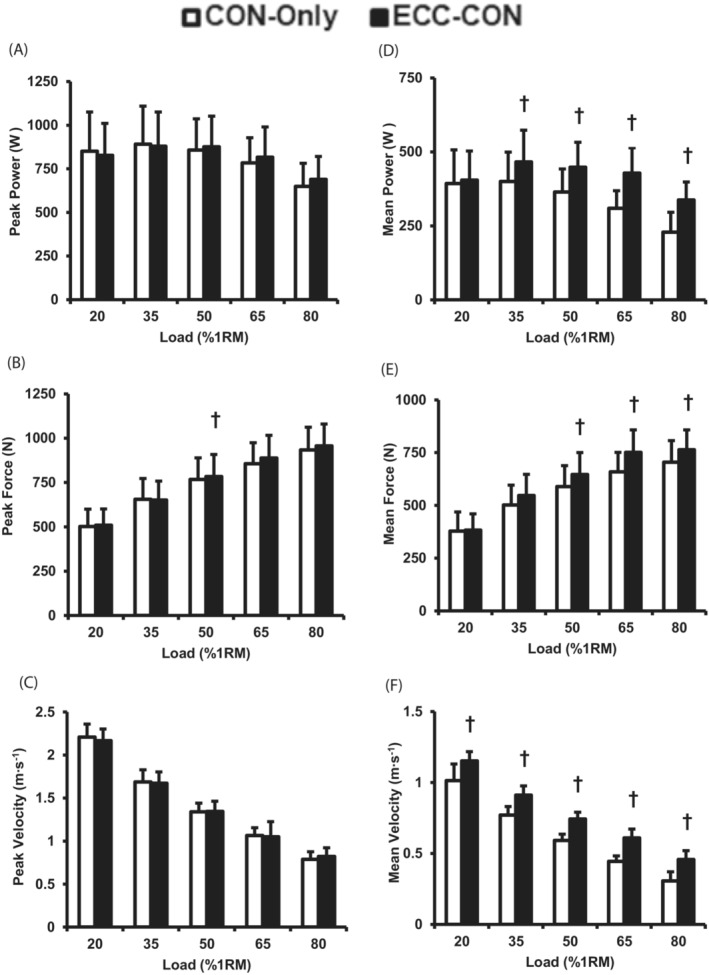
Peak and mean power (A, D) force (B, E) and velocity (C, F) during the concentric phase of concentric‐only (CON‐Only) and eccentric‐concentric (ECC‐CON) contractions with a range of five loads (20–80% 1RM). Data are presented as mean ± SD. † significantly (*p* < 0.05) greater than CON‐Only.

**FIGURE 3:**
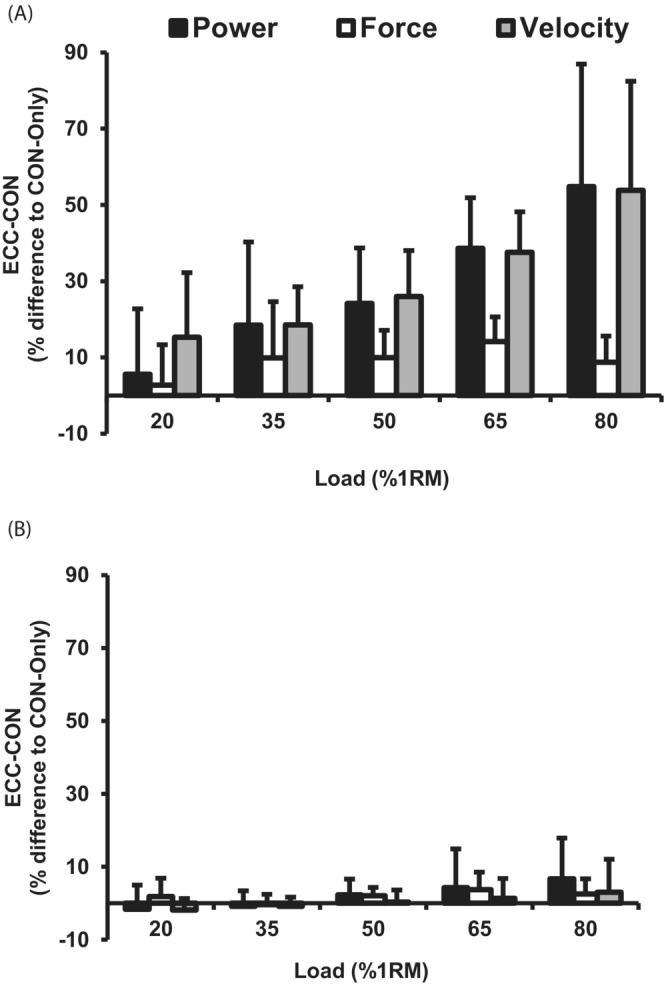
Percentage differences between eccentric‐concentric (ECC‐CON) versus concentric‐only (CON‐ONLY) contractions for a range of five loads (20–80% 1RM) (positive values, ECC‐CON > CON‐Only). (A) mean power, force, and velocity, (B) peak power, force and, velocity. Data are presented as mean ± SD.

A prior eccentric lowering phase produced higher concentric mean power, mean force, and mean velocity (two‐way ANOVA, main effects for contraction type, all *p* < 0.001 Figure 2D–F). Furthermore, for mean power, mean force and mean velocity during the concentric phase there was a significant main effect of load (two‐way ANOVA, all *p* < 0.001), and for mean power and mean force there was a significant load × type of contraction interaction effect (two‐way ANOVA, all *p* < 0.004), but this was not the case for mean velocity (*p* = 0.663). Subsequent post hoc analysis revealed that ECC‐CON produced higher mean power than CON‐Only for most (35–80% 1RM, paired *t*‐test, all *p* < 0.031, ∆ = 19 to 55%, ES = 0.6–1.7 “Moderate to Large”), but not all loads (20% 1RM, paired *t*‐test, *p* = 0.53). Mean force was greater for ECC‐CON than CON‐Only for moderate to heavy loads (50–80% 1RM; paired *t*‐test, all *p* < 0.003, ∆ 9 to 14%, ES = 0.6–0.9 “Moderate to Large”), but not lighter loads (20–35% 1RM, paired *t*‐test, all *p* > 0.05). Whereas mean velocity was greater for ECC‐CON versus CON‐Only for all loads (paired *t*‐test, all *p* < 0.005, ∆ 15 to 54%, ES = 1.5–3.3 “Large”). Figure 3 highlights the percentage differences between CON‐Only and ECC‐CON for mean and peak power, force and velocity variables. As load increased ECC‐CON had progressively greater percentage superiority to CON‐Only for mean power (∆6 to 55%) and mean velocity (∆15 to 54%; Figure 3).

For concentric peak power and peak velocity, there was no main effect of a prior eccentric lowering phase (i.e., type of contraction) or an interaction effect (i.e., load × type of contraction; two‐way ANOVA, *p* > 0.052, Figure 2A,C), whereas for peak force during the concentric phase, there was a main effect of type of contraction and an interaction effect (two‐way ANOVA, *p* < 0.033 Figure 2B), and post‐hoc analysis revealed no difference in peak force for the majority of the loads (i.e., 20–35 and 65–80% 1RM, paired *t*‐test, all *p* > 0.140), but a modestly higher peak force at 50% 1RM only (paired *t*‐test, *p* = 0.047, ∆ = 9%, ES = 0.13 “Small”). There was also a significant main effect of load for peak power, force, and velocity (two‐way ANOVA, all *p* < 0.001). There were relatively small percentage differences between CON‐Only and ECC‐CON for peak power, force, and velocity (Figure 3B).

### 3.3 Muscle activation during the whole concentric phase

**FIGURE 4:**
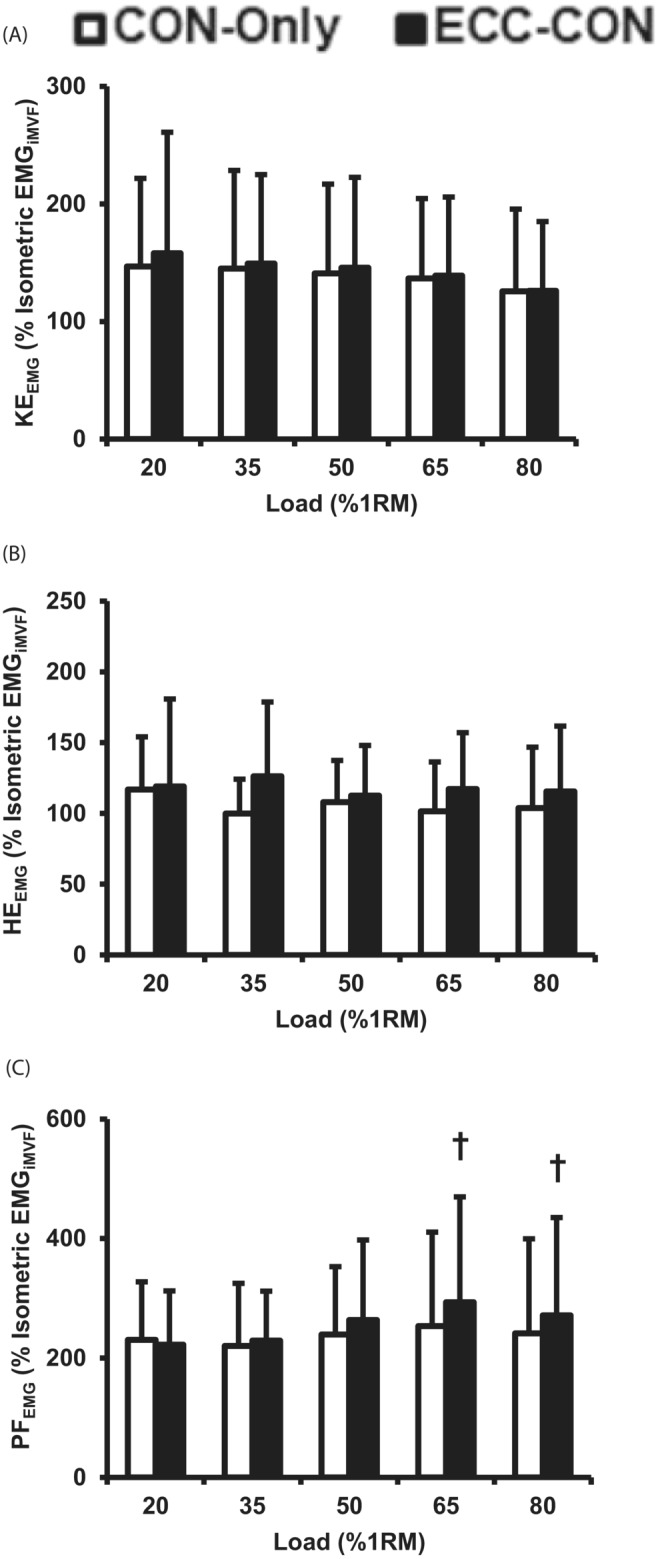
Surface electromyography amplitude (normalized to EMG amplitude at iMVF) for the knee extensors (A, KE_EMG_), hip extensors (B, HE_EMG_), and plantar‐flexors (C, PF_EMG_) during the concentric phase of concentric‐only (CON‐Only) and eccentric‐concentric (ECC‐CON) contractions with a range of five loads (20–80% 1RM). Data are presented as mean ± SD. † significantly (*p* < 0.05) greater than CON‐Only.

When EMG was assessed over the concentric phase, there was no main effect of contraction type or interaction effect on KE_EMG_ (two‐way ANOVA, *p* > 0.057, Figure 4A), whereas there was a significant main effect of load for KE_EMG_ (two‐way ANOVA, *p* = 0.015, Figure 4A). There was also no main effect of contraction type, load, or interaction effect for HE_EMG_ with similar HE_EMG_ reported during both ECC‐CON and CON‐Only (two‐way ANOVA, *p* > 0.066, Figure 7B). There was, however, a main effect of contraction type and an interaction effect on PF_EMG_ (two‐way ANOVA, all *p* < 0.013, Figure 4C), with further analysis revealing that PF_EMG_ was greater during ECC‐CON than CON‐ONLY for heavy loads (65–80% 1RM, paired *t*‐test, all *p* < 0.005, ∆ = 12 to 15%, ES = 0.18–0.24 “Small”), but not light/moderate loads (20–50% 1RM, all *p* > 0.05). There was no main effect of load for PF_EMG_ (two‐way ANOVA, all *p* = 0.278).

### 3.4 Neuromechanical differences during the percentage of contraction duration

**FIGURE 5:**
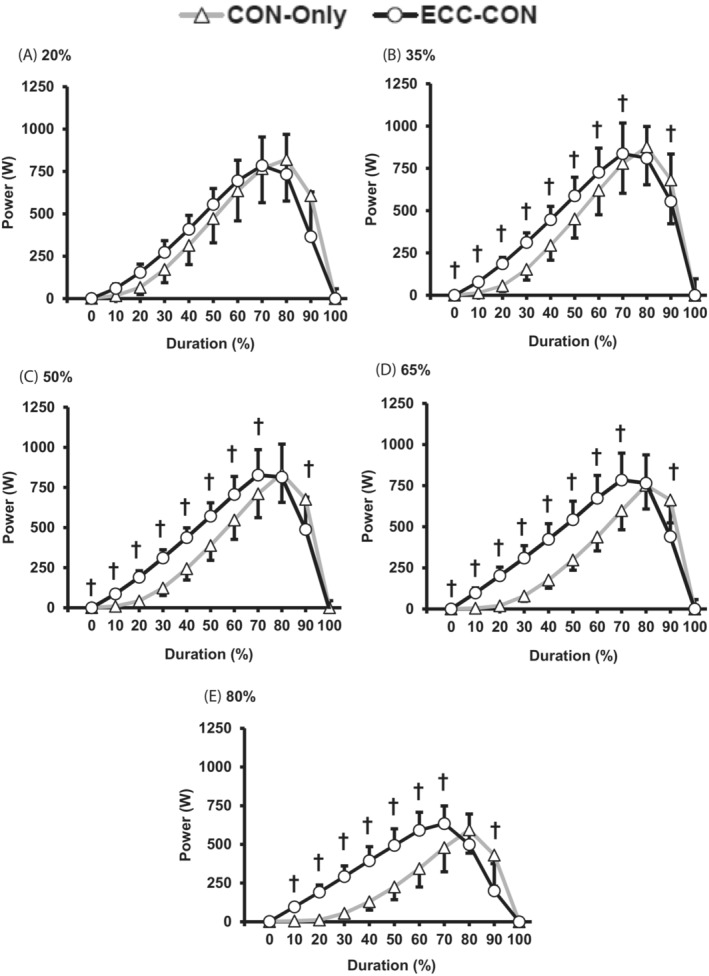
Power throughout (movement duration %) the concentric phase of concentric‐only (CON‐Only) and eccentric‐concentric (ECC‐CON) contractions at each of five different loads (A–E; 20–80% 1RM). Data are presented as mean ± SD. † ECC‐CON significantly (*p* < 0.05) different to CON‐Only.

**FIGURE 6:**
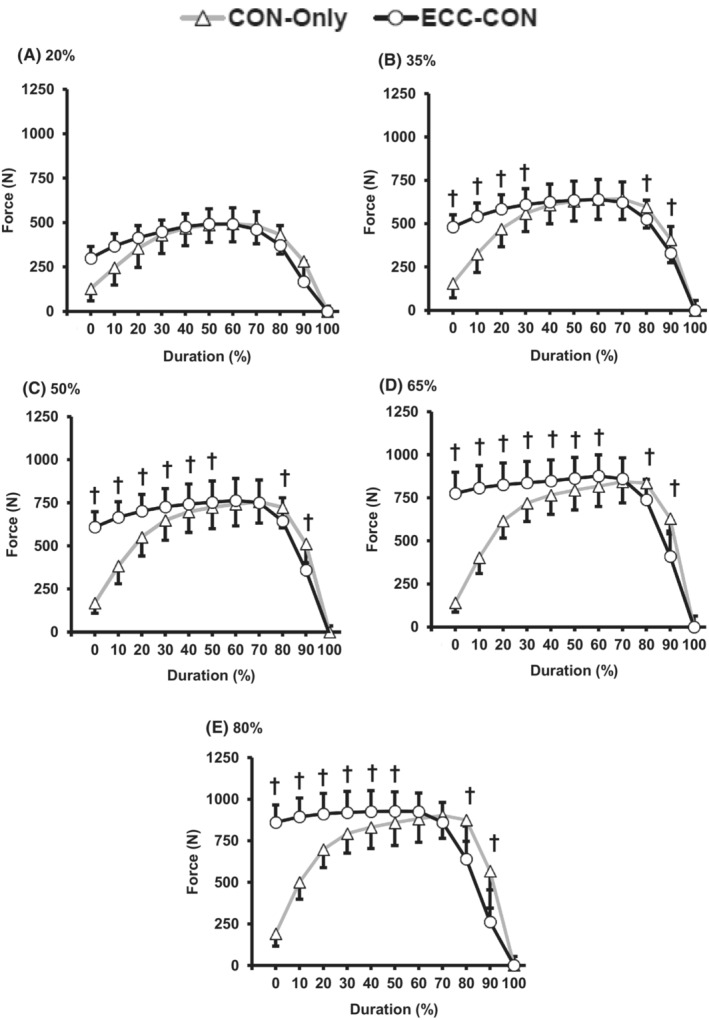
Force production throughout (% duration) the concentric phase of concentric‐only (CON‐Only) and eccentric‐concentric (ECC‐CON) contractions at each of five different loads (A–E; 20–80% 1RM). Data are presented as mean ± SD. † ECC‐CON was significantly (*p* < 0.05) different to CON‐Only.

**FIGURE 7:**
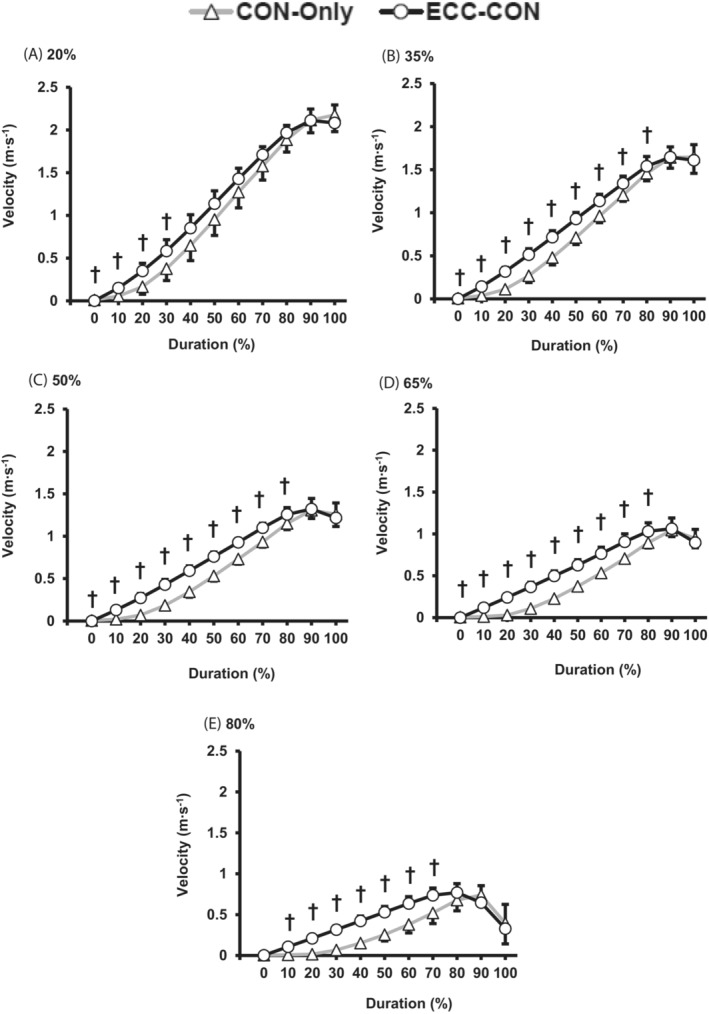
Velocity throughout (movement duration %) the concentric phase for concentric‐only (CON‐Only) and eccentric‐concentric (ECC‐CON) contractions at each of five different loads (A–E; 20–80% 1RM). Data are presented as mean ± SD. † ECC‐CON was significantly (*p* < 0.05) different to CON‐Only.

For a majority of loads (35–80% 1RM) power was higher for ECC‐CON versus CON‐Only for most of the contraction duration (10 to 70% of contraction duration, Figure 5B–E). Similarly, for the majority of loads (i.e., >20% 1RM) ECC‐CON produced greater force than CON‐Only for the most of contraction duration (0 to 60% contraction duration; Figure 6B–E). The differences in velocity between ECC‐CON versus CON‐Only also extended throughout a greater proportion of the concentric contraction as load increased; from only the early phase of contraction with the lightest load (20% 1RM, 0 to 30% of contraction duration) up to 0 to 70% of contraction duration for all other loads (35–80% 1RM, Figure 7A–F).

There was no difference in KE_EMG_ measured at different percentages of contraction duration across all loads (Supplementary Material—Figure S1). For heavier RT loads, ECC‐CON produced greater HE_EMG_ than CON‐Only at some points during the first half of the contractions (65% 1RM, 0–10% and 30–40% contraction duration; 80% 1RM, 0–50% contraction duration), but this was reversed at some points during the later stages of contraction with HE_EMG_ of CON‐Only exceeding ECC‐CON (65% 1RM, 80–100% contraction duration; 80% 1RM, 80% contraction duration; Supplementary Material—Figure S2). Finally, PF_EMG_ was greater for ECC‐CON than CON‐Only during the middle part of the contraction for 65% 1RM (30 to 60% contraction duration) and 80% 1RM (20 to 60% contraction duration, Supplementary Material—Figure S3).

## 4 DISCUSSION

The purpose of this study was to compare the presence and absence of a prior eccentric lowering phase of contraction on the concentric neuromechanics (power, force, velocity, sEMG) of older adults lifting a range of loads. The main findings supported our hypothesis as a prior eccentric phase increasingly enhanced mean power during the concentric phase of contraction as load increased (+19 up to 55% for loads 35–80% 1RM). Similarly, mean velocity was consistently higher after a prior eccentric phase, and increasingly so as load increased (+15 to 54%), whereas mean force showed more modest differences only at the higher loads (+9 to 14%, 50–80% 1RM) and there were no consistent differences in muscle activation. The greater mean power, force and velocity performance after a prior eccentric contraction was due to the higher force values at the start of concentric phase, which led to higher velocities and, thus, also greater power typically throughout most of the contraction duration. Despite this quicker development of concentric power after a prior eccentric contraction, peak power, which typically occurred late in the concentric phase of contraction was unaffected by a prior eccentric contraction. Overall, a prior eccentric phase of contraction enhanced mean, but not peak, power and velocity performance due to elevated force during the early phase of concentric contraction in older adults and would appear to provide a greater stimulus for power adaptations with regular RE.

This current study provides a detailed comparison of ECC‐CON and CON‐Only contractions, for the first time in older adults to inform the optimization of power RE for this population. It involved a comprehensive assessment of neuromechanical variables (i.e., mean, peak, and throughout the contraction measurements of force, velocity, power, and activation), over a wide range of loads typically used for resistance training and, thus, eliminated some limitations of previous studies of younger groups (e.g., less comprehensive measurements and/or a smaller range of loads).^17^, ^36^ The values generated in the current study were broadly similar to the peak power, force, and velocity values previously reported for young and older adults during isoinertial leg press contractions.^10^, ^17^, ^37^

Our finding of greater mean (∆ 6 to 55% across loads), but not peak (∆ −2 to +7% across loads) concentric power immediately after a prior eccentric action, compared with CON‐Only, in older adults is consistent with previous reports in young adults of higher mean power performance but no difference in peak power performance.^15^ Mean power, as the average rate of performing mechanical work during the whole movement may provide the more meaningful metric of whole movement function, whereas peak power is based on just one instant that in this task occurs relatively late in the movement. Hence, we consider the augmentation of mean power by a prior eccentric action the more important and functionally relevant outcome. In the current study, the augmentation of mean power was a remarkable 55% at the highest load, which is a large effect compared with previous studies in young adults.^14^, ^15^ Older adults have been found to have particularly compromised concentric function perhaps in part due to shortening‐induced force depression,^31^ whereas their eccentric function is well preserved^32^ perhaps due to RFE.^24^ Given the pronounced difference in eccentric and concentric function in older adults, their concentric function may be particularly sensitive to a prior eccentric contraction, and it is recommended that future work compare the benefits of a prior eccentric contraction in young and older adults. The underpinning force and velocity variables showed a similar pattern with greater concentric mean velocity at all loads (∆ 15 to 54%) and mean force at most loads (∆ 3 to 14%) after a prior eccentric phase, but similar peak velocity (∆ −2 to +3%) and force (∆ −0.4 to +4%). Therefore, the findings of this study taken together with the existing evidence demonstrates that a prior eccentric lowering phase augments mean concentric power, velocity and force performance. Hence, the inclusion of a prior eccentric lowering phase appears appealing for those prescribing RE for older adults looking to improve/maintain power.

Considering concentric mean power, the advantageous effect of a prior eccentric contraction increased with load, being 6, 19, 24, 39, and 55% greater with each of the five ascending loads from 20 to 80% 1RM (Figure 3). This load dependence of the ECC‐CON power enhancement appeared to be primarily due to a similar load‐related effect of ECC‐CON on mean velocity (15, 19, 26, 38 and 54% greater than CON‐Only for ascending loads), rather than the more limited enhancement of mean force (3, 10, 10, 14, and 9% greater than CON‐Only for ascending loads). This benefit of a prior eccentric contraction increasing with load appears to be a novel finding given that previous research has tended to find a relatively consistent enhancement of concentric power, force, velocity, and muscle activation irrespective of RT load.^14^, ^15^, ^19^ However, this may have been due to lighter loads being lowered faster than heavy loads in previous studies (eccentric duration at 80% 1RM = 1.23 vs. 0.64 s at 40% 1RM^19^; 15% 1RM at ~0.9 m·s^−1^ vs. 100% 1RM at ~0.25 m·s^−1^, Ref. [15]) as eccentric lowering velocity has been found to independently influence concentric power, force, and velocity performance.^13^, ^17^, ^38^ Faster eccentric velocities require greater force production in the late eccentric phase to decelerate the load, which facilitates a greater initial concentric force and, thus, greater accelerations during the early stages of concentric motion. Hence, in the current study, eccentric lowering velocity was controlled, as was concentric range of motion, to standardize these potential confounders.^25^ Therefore, standardizing the eccentric lowering velocity across loads appears to have better isolated the effect of load on the enhancement of mean concentric power, force, and velocity after an eccentric phase. Overall, both load (current study) and lowering velocity,^13^ appear to affect the enhancement of power gained by a prior eccentric contraction.

The similarity of peak power, force, and velocity between ECC‐CON and CON‐Only was despite clear differences in instantaneous power, force, and velocity throughout at least the first half of the concentric movement for power, force, and velocity at moderate to heavy loads (0 to 50% movement duration; Figures 5, 6, 7), and the early phase of the concentric movement for velocity at light loads (0 to 30% movement duration). However, peak power, velocity, and in most cases peak force occurred relatively late in the concentric movement (i.e., >70% of movement duration), by which stage the mechanical advantages of a prior eccentric contraction were no longer present.^17^ Thus, it appears that when ECC‐CON contractions reach the latter phase of the concentric movement (>70% of movement duration) the benefit of a prior eccentric phase on concentric muscle performance had diminished sufficiently to be undetectable.

There are several potential mechanisms, including connective tissue energy storage and release, pre‐activation, stretch reflex, and sarcomere level RFE, which could explain the greater concentric mean power, force, and velocity performance during ECC‐CON than CON‐Only. The findings of this current study are not able to fully delineate the role of these mechanisms; however, the similarity in concentric phase muscle activation (overall or in general during the early part of the concentric phase) between ECC‐CON and CON‐Only suggests no substantial stretch reflex effect on activation.^26^ Pre‐activation during the eccentric phase did elevate force production at the onset of concentric action (i.e., 0% of movement duration) for all loads ≥35% 1RM, and force at onset also increased with load (~300 N at 20% 1RM to ~900 N at 80% 1RM). These higher forces at onset during ECC‐CON appear to have led to consistently higher force during the early concentric phase of ECC‐CON versus CON‐Only (e.g., at least 0–30% contraction duration for loads ≥35% 1RM) and subsequently to elevated instantaneous power throughout most of the concentric movement (i.e., 0–70% of movement duration, Figure 5B–E). Since the concentric phase of both types of contractions started at zero velocity, it was the elevated force values during ECC‐CON that led to the greater acceleration,^17^ and subsequently higher velocities during ECC‐CON than CON‐Only for a significant proportion of the concentric movement (0–70% movement duration, 20–80% 1RM, Figure 7A–F). Thus, pre‐activation and elevated active force at the onset of concentric action are a plausible mechanism for the augmentation of mean power after a prior eccentric action. Furthermore, from Figure 6, the force advantage of a prior eccentric phase at concentric movement onset clearly grows with load and likely explains why the augmentation of concentric mean power was greater at higher loads. Whilst connective tissue energy storage and release^23^, ^25^ along with sarcomere RFE^30^ are also well documented mechanisms for the benefits of a prior eccentric contraction the current study was not able to inform the roles of these mechanisms.

### 4.1 Limitations, practical and future applications

Based on the principle of training specificity, if the goal of an RE program is to improve the muscle power output of older adults, then contractions that provide the greatest muscle power output would seem to provide the ideal training stimulus. The findings of this study showed that ECC‐CON contractions generated greater mean concentric power than CON‐Only and, therefore, may provide a superior training stimulus for power development. In practice, this supports the practice of performing RE lifts continuously without a rest/pause, and consequent unloading, between the eccentric and concentric contractions. Whilst this study provides information on how load and the presence of a prior eccentric lowering phase affected concentric power, force, and velocity during single repetitions, it was limited to the assessment of acute enhancements. Therefore, the chronic adaptations (e.g., functional, neural, and morphological) that may occur with prolonged ECC‐CON or CON‐Only training regimes remain to be explored in older adults. Furthermore, as noted the current study using an integrative multiple‐joint approach was not able to fully elucidate all the fundamental mechanisms with high precision (e.g., connective tissue energy storage and recoil). Finally, whilst many of the main effects in this study showed quite marked differences (e.g., in mean power and velocity) between ECC‐CON and CON‐Only, some of the other more subtle differences throughout the contraction or for peak measures may have become clearer with a larger number of participants and greater statistical power.

## 5 CONCLUSION

In conclusion, mean concentric power, force, and velocity were consistently higher after a prior eccentric contraction than without, and, therefore, appear to provide a greater RE stimulus for older adults looking to improve/maintain power. The higher force values during the initial phase of concentric contraction after a prior eccentric contraction appeared to lead to enhanced mean concentric velocity and power, although surprisingly peak concentric force, velocity, and power were unaffected by a prior eccentric contraction, likely as they occurred late in the contraction after any advantage of the prior eccentric contraction had subsided. This study also found a clear influence of the RE load on the augmentation of concentric performance, particularly power and velocity, by a prior eccentric contraction, with the largest benefits occurring at the highest loads.

## 6 PERSPECTIVE

The critical importance of power for healthy aging necessitates the optimisation of RE guidelines for power development. Therefore, understanding whether the inclusion of a prior eccentric lowering phase produces greater power performance than CON‐Only contractions in older adults warranted investigation. The main findings of this current study were that a prior eccentric lowering phase produced greater mean power (+19 to 55%) and velocity (+15 to 54%) performance for most RE loads, that is, 35–80% 1RM, with the magnitude of the difference in power and velocity performance increasing as load increased. Therefore, the performance of ECC‐CON contractions as opposed to CON‐Only contractions may offer a more potent power development stimulus for older adults looking to maintain/improve power performance. However, the potential for ECC‐CON contractions to improve muscle power in older adults more that CON‐Only following regular RE programme remains to be explored.

## FUNDING INFORMATION

Part of this study was supported by the Versus Arthritis Centre for Sport, Exercise, and Osteoarthritis Research (Grant reference: 21595).

## CONFLICT OF INTEREST STATEMENT

The authors declare that there is no conflict of interest, that no companies or manufacturers will benefit from the results of the study, and that the results of the study are presented clearly, honestly, and without fabrication, falsification, or inappropriate data manipulation.

## Supporting information


**Figure S1** Surface electromyography amplitude (normalized to EMG amplitude at iMVF) for the knee extensors throughout (movement duration %) the concentric phase of concentric‐only (CON‐Only) and eccentric‐concentric (ECC‐CON) contractions at each of five different loads (A–E; 20–80% 1RM). Data are presented as mean ± SD. There were no differences in EMG at any point during the concentric phase of CON‐Only and ECC‐CON.
**FIGURE S2** Surface electromyography amplitude (normalized to EMG amplitude at iMVF) for the hip extensors throughout (movement duration %) the concentric phase of concentric‐only (CON‐Only) and eccentric‐concentric (ECC‐CON) contractions at each of five different loads (A–E; 20–80% 1RM). Data are presented as mean ± SD. † ECC‐CON significantly (*p* < 0.05) different to CON‐Only.
**FIGURE S3** Surface electromyography amplitude (normalized to EMG amplitude at iMVF) for the plantar flexors throughout (movement duration %) the concentric phase of concentric‐only (CON‐Only) and eccentric‐concentric (ECC‐CON) contractions at each of five different loads (A–E; 20–80% 1RM). Data are presented as mean ± SD. † ECC‐CON significantly (*p* < 0.05) different to CON‐Only.

## Data Availability

The data that support the findings of this study are available from the corresponding author upon reasonable request.
