# Supplementary material for: The effect of a prior eccentric lowering phase on concentric neuromechanics during multiple joint resistance exercise in older adults
Source: Scand J Med Sci Sports. 2023 Jun 22;33(10):2009–24. doi: 10.1111/sms.14435 (PMC10946755; doi:10.1111/sms.14435)
Supplement: Supplementary file 1 — Figure S1 Surface electromyography amplitude (normalized to EMG amplitude at iMVF) for the knee extensors throughout (movement duration %) the concentric phase of concentric‐only (CON‐Only) and eccentric‐concentric (ECC‐CON) contractions at each of five different loads (A–E; 20–80% 1RM). Data are presented as mean ± SD. There were no differences in EMG at any point during the concentric phase of CON‐Only and ECC‐CON. FIGURE S2 Surface electromyography amplitude (normalized to EMG amplitude at iMVF) for the hip extensors throughout (movement duration %) the concentric phase of concentric‐only (CON‐Only) and eccentric‐concentric (ECC‐CON) contractions at each of five different loads (A–E; 20–80% 1RM). Data are presented as mean ± SD. † ECC‐CON significantly (p < 0.05) different to CON‐Only. FIGURE S3 Surface electromyography amplitude (normalized to EMG amplitude at iMVF) for the plantar flexors throughout (movement duration %) the concentric phase of concentric‐only (CON‐Only) and eccentric‐concentric (ECC‐CON) contractions at each of five different loads (A–E; 20–80% 1RM). Data are presented as mean ± SD. † ECC‐CON significantly (p < 0.05) different to CON‐Only. [file SMS-33-2009-s001.docx]

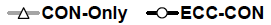


**D. 65%**

**C. 50%**

**A. 20%**

**B. 35%**

**E. 80%**

**Supplementary Material, Figure S1.** Surface electromyography amplitude (normalised to EMG amplitude at iMVF) for the knee extensors throughout (movement duration %) the concentric phase of concentric-only (CON-Only) and eccentric-concentric (ECC-CON) contractions at each of five different loads (A - E; 20 - 80 %1RM). Data are presented as mean ± SD. There were no differences in EMG at any point during the concentric phase of CON-Only and ECC-CON.

 **Supplementary Material, Figure S2.** Surface electromyography amplitude (normalised to EMG amplitude at iMVF) for the hip extensors throughout (movement duration %) the concentric phase of concentric-only (CON-Only) and eccentric-concentric (ECC-CON) contractions at each of five different loads (A - E; 20 - 80 %1RM). Data are presented as mean ± SD. † ECC-CON significantly (P < 0.05) different to CON-Only.

**Supplementary Material, Figure S3.** Surface electromyography amplitude (normalised to EMG amplitude at iMVF) for the plantar flexors throughout (movement duration %) the concentric phase of concentric-only (CON-Only) and eccentric-concentric (ECC-CON) contractions at each of five different loads (A - E; 20 - 80 %1RM). Data are presented as mean ± SD. † ECC-CON significantly (P < 0.05) different to CON-Only.
